# How to Approach Para-Aortic Lymph Node Metastases During Exploration for Suspected Periampullary Carcinoma: Resection or Bypass?

**DOI:** 10.1245/s10434-020-08304-0

**Published:** 2020-03-10

**Authors:** Bobby K. Pranger, Dorine S. J. Tseng, Sander Ubels, Hjalmar C. van Santvoort, Vincent B. Nieuwenhuijs, Koert P. de Jong, Gijs Patijn, I. Quintus Molenaar, Joris I. Erdmann, Vincent E. de Meijer

**Affiliations:** 1 Department of Hepatopancreatobiliary Surgery and Liver Transplantation, University Medical Center Groningen, University of Groningen, Groningen, The Netherlands; 2 Department of Hepatopancreatobiliary Surgery, Regional Academic Cancer Center Utrecht, St. Antonius Hospital Nieuwegein and University Medical Center Utrecht, Utrecht, The Netherlands; 3 Department of Surgery, Isala Clinics Zwolle, Zwolle, The Netherlands; 4 Department of Hepatopancreatobiliary Surgery, University Medical Center Utrecht, Utrecht, The Netherlands

## Abstract

**Background:**

Intraoperative para-aortic lymph node (PALN) sampling during surgical exploration in patients with suspected pancreatic head cancer remains controversial.

**Objective:**

The aim of this study was to assess the value of routine PALN sampling and the consequences of different treatment strategies on overall patient survival.

**Methods:**

A retrospective, multicenter cohort study was performed in patients who underwent surgical exploration for suspected pancreatic head cancer. In cohort A, the treatment strategy was to avoid pancreatoduodenectomy and to perform a double bypass procedure when PALN metastases were found during exploration. In cohort B, routinely harvested PALNs were not examined intraoperatively and pancreatoduodenectomy was performed regardless. PALNs were examined with the final resection specimen. Clinicopathological data, survival data and complication data were compared between study groups.

**Results:**

Median overall survival for patients with PALN metastases who underwent a double bypass procedure was 7.0 months (95% confidence interval [CI] 5.5–8.5), versus 11 months (95% CI 8.8–13) in the pancreatoduodenectomy group (*p* = 0.049). Patients with PALN metastases who underwent pancreatoduodenectomy had significantly increased postoperative morbidity compared with patients who underwent a double bypass procedure (*p* < 0.001). In multivariable analysis, severe comorbidity (ASA grade 2 or higher) was an independent predictor for decreased survival in patients with PALN involvement (hazard ratio 3.607, 95% CI 1.678–7.751; *p* = 0.001).

**Conclusion:**

In patients with PALN metastases, pancreatoduodenectomy was associated with significant survival benefit compared with a double bypass procedure, but with increased risk of complications. It is important to weigh the advantages of resection versus bypass against factors such as comorbidities and clinical performance when positive intraoperative PALNs are found.

**Electronic supplementary material:**

The online version of this article (10.1245/s10434-020-08304-0) contains supplementary material, which is available to authorized users.

Pancreatic cancer is one of the leading causes of cancer-related death in developed countries, and its incidence is increasing.^1^ Although progress has been made in treatment options, the 5-year survival rate remains poor regardless of disease state.^2^ Pancreatoduodenectomy is the only potential curative treatment option for patients with suspected pancreatic ductal adenocarcinoma (PDAC). Despite the curative treatment options, PDAC should be considered a systemic disease and recurrence is inevitable in most patients. Even after pancreatoduodenectomy with curative intent, the median survival for patients with PDAC is poor, at only 18 months.

It is clear that major surgery for limited or no survival benefit should be avoided. Optimal preoperative and perioperative staging is therefore essential. The decision to perform a pancreatoduodenectomy currently depends on vascular involvement, distant metastases and lymph node metastases.^3^^,^^4^ Lymph node involvement of the para-aortic lymph nodes (PALNs) corresponds with distant metastases according to the Japanese Pancreas Society Classification of Pancreatic Cancer,^5^ but preoperative evaluation of these extraregional lymph nodes is difficult because the accuracy of diagnostic imaging is limited.^6^ Several studies suggest a poor survival for patients who underwent pancreatoduodenectomy with positive PALNs compared with patients in whom PALNs were negative. Therefore, it was suggested that PALN involvement is a contraindication to pancreatoduodenectomy and PALN sampling with frozen section examination should be performed routinely.^7^^–^10 Other studies have identified patients who might benefit from palliative resection and have shown that there are long-term survivors in this population.^11^^,^^12^ Based on these data, the International Study Group on Pancreatic Surgery (ISGPS) did not reach consensus regarding routine resection of PALNs, due to variation in the literature and different expert opinions.^13^

The consequences of detecting intraoperative PALN metastases are unclear. All the previously performed studies included cohorts with patients who underwent pancreatoduodenectomy. None of the aforementioned studies evaluated the outcomes of patients with positive PALNs who did not undergo resection. When positive PALNs or other contraindications for resection are encountered during exploration, a palliative (double) bypass procedure is typically performed. This procedure is also associated with considerable morbidity.^14^

The aim of this study was to compare pancreatoduodenectomy with a palliative double bypass in patients with PDAC in whom PALNs were routinely sampled during surgical exploration.

## Methods

### Patients and Outcomes

**Fig. 1:**
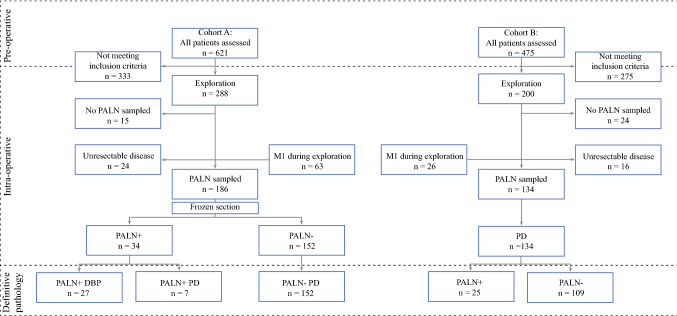
Treatment strategies of patients in cohorts A and B with potentially resectable pancreatic head cancer who underwent surgical exploration and PALN sampling with or without immediate pathological frozen section analysis. *PALN* para-aortic lymph node, *PD* pancreatoduodenectomy, *DBP* double bypass procedure

A multicenter, retrospective analysis was performed of all patients undergoing pancreatoduodenectomy for suspected PDAC at the University Medical Center Groningen, Groningen, The Netherlands, between January 2004 and December 2016 (Cohort A), and the University Medical Center Utrecht, Utrecht, and Isala Clinics Zwolle, Zwolle, The Netherlands, between January 2013 and December 2016 (combined in Cohort B). Patients were retrospectively identified using the prospectively maintained databases for pancreatic cancer registration. This study was approved by the Ethics Committee of the University Medical Center Groningen (METc 201500644). A preoperative multiphase computed tomography (CT) scan was routinely used to investigate potential vascular involvement and/or distant metastases. In accordance with the ISGPS, clinical suspicion of PDAC was sufficient to proceed to explorative laparotomy or pancreatic resection, and histopathological evidence was not mandatory. In Cohort A, all patients underwent routine PALN sampling. If perioperative frozen section analysis revealed lymph node metastasis, the typical strategy was to abort resection with curative intent and perform a palliative double bypass procedure on indication; however, in selected patients, a pancreatoduodenectomy was performed. In contrast, in Cohort B, all patients underwent pancreatoduodenectomy, and PALNs were routinely harvested and examined only with the final resection specimen, not with perioperative frozen section analysis (Fig. 1).

Patients with neuroendocrine tumors, benign cysts and pancreatitis were excluded (verified by histopathology) post hoc. Furthermore, patients with R2 resection were also excluded from both cohorts. The primary outcome was overall survival. The survival status of all patients was assessed on 30 March 2018, using the Dutch Municipal Personal Records Database.

### Surgical Procedures

A standard pancreatoduodenectomy was performed in a similar fashion in all centers. With routine PALN sampling, the fatty tissue from the aortocaval window, i.e. all tissue between the aorta and inferior vena cava from the inferior border of the left renal vein to the cranial border of the inferior mesenteric artery was harvested (lymph node station 16b1).^15^ All patients followed standardized preoperative and postoperative treatment protocols. All procedures were performed or supervised by an experienced hepatopancreatobiliary surgeon.

### Complications

All complications within a 90-day period after operation were classified according to the Clavien–Dindo classification.^16^ After grading each complication, the Comprehensive Complication Index (CCI) was calculated for each patient to determine the overall severity of all complications combined.^17^ The CCI was calculated by using the CCI calculator tool available at www.assessurgery.com. The final index yields a score from 0 (no complication) to 100 (death).^17^

### Statistics

Continuous data are expressed as medians with interquartile ranges (IQRs), and categorical variables are expressed as numbers with percentages. Variables were compared using the Mann–Whitney *U* test, Chi square test and Fisher’s exact test, where appropriate. Survival analyses were performed using Kaplan–Meier analysis and the log-rank test. Univariable and multivariable analyses were performed using Cox proportional hazards regression, calculating hazard ratio (HR), after checking proportional hazards assumption. Relevant demographical, clinical, and pathological variables were selected for multiple Cox regression analysis. Patients were censored at the time of death. A sensitivity analysis was performed for patients with PDAC and who received adjuvant chemotherapy. Missing values were labeled as user-missing values and were excluded from statistical analysis by pairwise deletion. A *p* value < 0.05 was considered statistically significant. All statistical analyses were performed using SPSS version 23.0 (SPSS Inc., Chicago, IL, USA).

## Results

### Patient Characteristics

A total of 1096 patients were assessed for study eligibility. In cohort A, 288 patients with suspected pancreatic head cancer underwent exploration. During exploration, 24 patients were found to have unresectable disease due to advanced vascular involvement, 63 patients had metastatic disease, and in 15 patients, no PALN sampling was performed due to the absence of lymph nodes or technical difficulties. The remaining 186 patients underwent PALN sampling with frozen section analysis. Patients with negative PALNs underwent pancreatoduodenectomy (*n* = 152). In one patient, exploration was aborted because of a false-positive frozen section. This patient underwent pancreatoduodenectomy 2 weeks later when definitive pathology showed no PALN involvement. In patients with positive PALNs, resection with curative intent was aborted and a palliative double bypass procedure was performed (*n* = 27). A total of seven patients underwent resection despite intraoperative positive frozen section analysis of PALNs. The decision to continue resection was based on the patient’s age and clinical performance score. In two patients, the decision to continue exploration was partially based on false-negative frozen sections (Fig. 1).

In cohort B, 200 patients with suspected pancreatic head cancer underwent exploration. During exploration, 16 patients were found to have unresectable disease, 26 patients had metastatic disease, and in 24 patients, no PALN sampling was performed due to the absence of lymph nodes or technical difficulties. The remaining 134 patients underwent pancreatoduodenectomy with PALN sampling, but without frozen section analysis. After definitive pathological examination, positive PALNs were found in 25 (19%) patients (Fig. 1).

Overall, both cohorts had similar clinical characteristics, except for distribution of the American Society of Anesthesiologists (ASA) scores, which differed slightly (*p* = 0.027) (Table 1).

**Table 1:** Clinical characteristics of patients who underwent surgery for pancreatic head cancer, stratified by cohort

Characteristic	Cohort A [*n* = 186]	Cohort B [*n* = 134]	*p* value
Age at surgery, years [median (IQR)]	68 (61–74)	66 (60–72)	0.099
Sex, males	96 (51.6)	75 (56.0)	0.441
ASA fitness grade			**0.027**
Class I	22 (11.8)	31 (23.1)	
Class II	126 (67.7)	80 (59.7)	
Class III	38 (20.4)	23 (17.2)	
PALN+	34 (18.3)	25 (18.7)	0.932

Bold value indicates statistical significance

Data are expressed as *n* (%) unless otherwise specified

For comparison between two groups Mann–Whitney *U* test were used for continuous variables and for binary variables Chi squared test or Fisher’s exact test were used as appropriate

*ASA* American Society of Anesthesiologists, *PALN *+ positive para-aortic lymph nodes, *IQR* interquartile range

The pathological characteristics of patients who underwent pancreatoduodenectomy are summarized in Table 2. Because the proportion of patients diagnosed with PDAC in cohort A (60%) differed significantly from cohort B (73%; *p* = 0.009), the pathological characteristics of patients with PDAC were tabulated separately (Table 2). In cohort B, more positive resection margins (45%) were observed compared with cohort A (28%; *p* = 0.002), and more patients underwent adjuvant chemotherapy in cohort B (50%) compared with cohort A (23%; *p* < 0.001). This difference between cohorts remained significant for patients with PDAC (34 vs. 64%; *p* < 0.001), as well as for patients operated between 2013 and 2016 (42 vs. 64%; *p* = 0.025).

**Table 2:** Pathological characteristics of patients who underwent resection for pancreatic head cancer, stratified by cohort

Characteristic	All resections			PDAC only		
	Cohort A [*n* = 159]	Cohort B [*n* = 134]	*p* value	Cohort A [*n* = 96]	Cohort B [*n* = 98]	*p* value
Etiology [*n* (%)]			**0.009**	–	–	–
PDAC	96 (60.4)	98 (73.1)				
Distal cholangiocarcinoma	22 (13.8)	18 (13.4)				
Carcinoma of the papilla of Vater	38 (23.9)	13 (9.7)				
Other or not specified	3 (1.9)	5 (3.7)				
Tumor size [mean (SD)]	3.0 (1.2)	3.1 (1.2)	0.639	3.3 (1.1)	3.3 (1.1)	0.740
N + [*n* (%)]	110 (69.2)	102 (76.2)	0.186	77 (80.2)	80 (81.6)	0.801
Number of analyzed nodes [median (range)]	13 (2–38)	16 (3–52)	**0.004**	13 (2–38)	16 (3–52)	**0.027**
Lymph node ratio [median (IQR)]	0.12 (0–0.25)	0.18 (0.04–0.30)	0.063	0.18 (0.06–0.30)	0.18 (0.06–0.29)	0.993
Number of analyzed PALNs [median (IQR)]	2 (1–14)	2 (1–10)	0.950	2 (2–4)	2.5 (1–4)	0.737
Number of involved PALNs, *median (range)*	0 (0–2)	0 (0–4)	**< 0.001**	0 (0–2)	0 (0–4)	**0.017**
Lymph node ratio, PALNs [median (IQR)]	0 (0–0)	0 (0–0)	**< 0.001**	0 (0.0–0.0)	0 (0.0–0.0)	0.715
Perineural invasion^a^ [*n* (%)]	109 (68.6)	107 (85.6)	**0.001**	83 (86.5)	84 (91.3)	0.292
Angioinvasion^b^	86 (54.4)	89 (71.8)	**0.003**	58 (60.4)	68 (74.7)	**0.037**
Positive resection margins [*n* (%)]	44 (27.7)	60 (44.8)	**0.002**	41 (42.7)	51 (52.0)	0.139
Adjuvant chemotherapy [*n* (%)]	37 (23.3)	67 (50.0)	**< 0.001**	33 (34.4)	62 (63.3)	**< 0.001**

Bold values indicate statistical significance

For comparison between two groups, the Mann–Whitney *U* test was used for continuous variables, and the Chi square or Fisher’s exact tests were used for binary variables as appropriate

^a^Nine missing, cohort B

^b^One missing, cohort A; 10 missing, cohort B

*PALN* para-aortic lymph node, *PDAC* pancreatic ductal adenocarcinoma, *N*+ positive lymph node status, *SD* standard deviation, *IQR* interquartile range

### Resection or (Double) Bypass with Para-Aortic Lymph Node [PALN] Involvement?

**Fig. 2:**
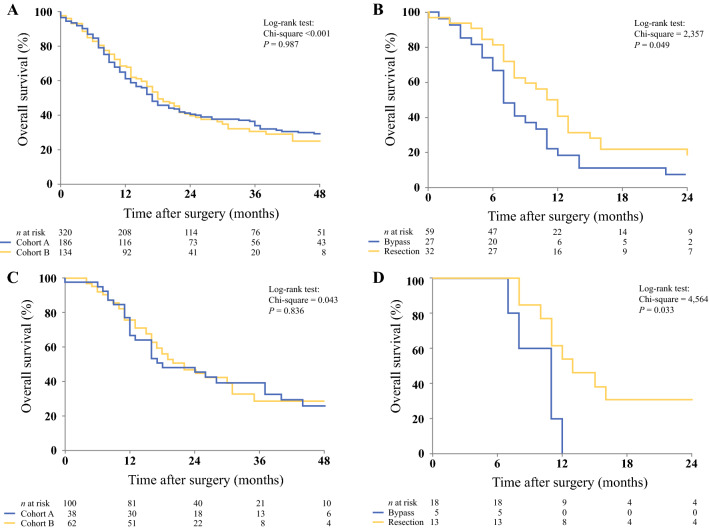
**a** Overall survival of all patients from cohorts A and B. The median overall survival in cohort A was 17 months (95% CI 13.9–20.1), compared with 18 months (95% CI 14.5–21.5) in cohort B (*p* = 0.987). **b** Overall survival of patients in cohorts A and B with PALN involvement who underwent bypass versus resection. Median survival was 7 months (95% CI 5.5–8.5) versus 11 months (95% CI 8.8–13.2; *p* = 0.049). **c** Overall survival of patients from cohorts A and B. All patients received postoperative chemotherapy. The median overall survival in cohort A was 24 months (95% CI 9.7–38.3), compared with 22 months (95% CI 15.3–28.7) in cohort B (*p* = 0.836). **d** Overall survival of patients in cohorts A and B with PALN involvement who underwent bypass versus resection. All patients received postoperative chemotherapy. Median survival was 11 months (95% CI 8.4–13.6) versus 13 months (95% CI 8.4–17.7; *p* = 0.033). *CI* confidence interval, *PALN* para-aortic lymph node

The median overall survival in cohort A was 17 months (95% CI 13.9–20.1), compared with 18 months (95% CI 14.5–21.5) in cohort B (*p* = 0.987) (Fig. 2a). The strategy concerning PALNs was different in both cohorts, but this did not yield a significant survival benefit for patients who underwent pancreatoduodenectomy. In total, 32 patients with PALN involvement underwent a pancreatoduodenectomy, whereas 27 patients underwent a double bypass procedure. The median survival was 11 months (95% CI 8.8–13.2) in patients who underwent pancreatoduodenectomy, compared with 7.0 months (95% CI 5.5–8.5) in patients who underwent a double bypass procedure instead of resection (*p* = 0.049) (Fig. 2b). In a sensitivity analysis regarding PDAC patients who received adjuvant chemotherapy, median overall survival for cohorts A and B was 24 months (95% CI 9.7–38.3) and 22 months (95% CI 15.3–28.7), respectively (*p* = 0.836) (Fig. 2c). In this subgroup, patients with PALN involvement had a median survival of 13 months (95% CI 8.3–17.7) after pancreatoduodenectomy (*n* = 13), compared with 11 months (95% CI 8.4–13.6) after a double bypass procedure (*n* = 5; *p* = 0.033) (electronic supplementary Table 1; Fig. 2d). Furthermore, there were significant differences in postoperative outcomes between both groups. More severe complications (Clavien–Dindo score of 3 or higher), a higher CCI score, and a longer time until discharge were seen in patients who underwent pancreatoduodenectomy with PALN involvement, when compared with patients who underwent a double bypass procedure (Table 3). A total of 14 (44%) patients who underwent pancreatoduodenectomy had severe complications, compared with two (7%) patients in the double bypass procedure group (*p* = 0.002). The median CCI scores were 34 (IQR 25.4–43.1) in the pancreatoduodenectomy group, compared with 8.7 (IQR 0–20.9) in the double bypass procedure group (*p* < 0.001). The median time until discharge was 12.5 days (IQR 10–18) and 9 days (IQR 8–12) for the pancreatoduodenectomy and double bypass procedure groups, respectively (*p* = 0.021). There was no significant difference in 30- and 90-day mortality (*p* = 0.588) (Table 3).

**Table 3:** Characteristics and postoperative outcomes of patients with positive para-aortic lymph nodes who underwent pancreatoduodenectomy versus a double bypass procedure

Characteristic	Resection (PD) [*n* = 32]	Bypass (DBP) [*n* = 27]	*p* value
Age at surgery, years [median (IQR)]	68 (61–75)	68 (57–73)	0.885
Sex, males	14 (43.8)	15 (55.6)	0.366
ASA fitness grade			0.768
Class I/II	27 (84.4)	31 (81.5)	
Class III	5 (15.6)	80 (18.5)	
Clavien–Dindo score of 3 or higher	14 (43.8)	2 (7.4)	**0.002**
CCI score [median (IQR)]	34.1 (25.4–43.1)	8.7 (0–20.9)	**< 0.001**
Time until discharge, days [median (IQR)]	12.5 (10–18)	9 (8–12)	**0.021**
Postoperative chemotherapy	13 (40.6)	5 (18.5)	0.066
30-day mortality	1 (3.1)	0 (0.0)	1.000
90-day mortality	1 (3.1)	2 (7.4)	0.588

Bold values indicate statistical significance

Data are expressed as *n* (%) unless otherwise specified

For comparison between two groups, the Mann–Whitney U test was used for continuous variables, and the Chi square or Fisher’s exact tests were used for binary variables

*ASA* American Society of Anesthesiologists, *CCI* Comprehensive Complication Index, *PD* pancreatoduodenectomy, *DBP* double bypass procedure, *IQR* interquartile range

### Survival of PALN Involvement Compared with Metastatic Disease during Exploratory Laparotomy

**Fig. 3:**
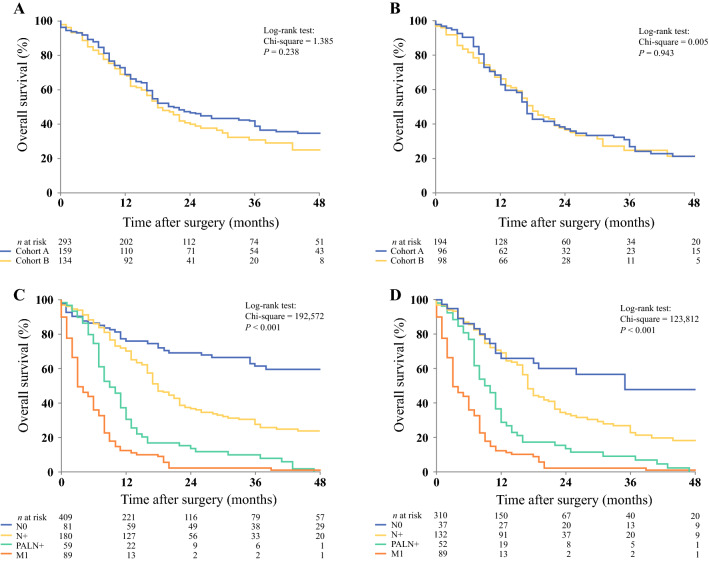
**a** Overall survival of all resections in cohort A versus cohort B. The median overall survival of all patients who underwent pancreatoduodenectomy in cohort A was 21 months (95% CI 15.6–26.4), compared with 18 months (95% CI 14.5–21.5) in cohort B (*p* = 0.238). **b** Overall survival of all resections with PDAC on final pathological examination of cohort A versus cohort B. The median overall survival after pancreatoduodenectomy for PDAC was 17 months (95% CI 15.0–19.0) versus 18 months (95% CI 14.7–21.3) in both cohorts (*p* = 0.943). **c** Overall survival of patients in cohorts A and B with negative lymph node (N0) status, positive lymph node (N +) status, PALN involvement (PALN +), and metastasized (M1) disease. Median overall survival was 65, 18, 9, and 3 months, respectively (*p* < 0.001). **d** Overall survival of patients in cohorts A and B with PDAC on final pathological examination, with N0, N+, PALN+, and M1 status. Median overall survival was 35, 17, 9, and 3 months, respectively (*p* < 0.001). *PDAC* pancreatic ductal adenocarcinoma, *PALN* para-aortic lymph node

The median overall survival of all patients who underwent pancreatoduodenectomy in cohort A was 21 months (95% CI 15.6–26.4), compared with 18 months (95% CI 14.5–21.5) in cohort B (*p* = 0.238) (Fig. 3a). For PDAC only, the median overall survival after pancreatoduodenectomy in cohort A was 17 months (95% CI 15.0–19.0) versus 18 months (95% CI 14.7–21.3) in cohort B, respectively (*p* = 0.943) (Fig. 3b). The survival of patients with PALN involvement was subsequently compared with the survival of patients without lymph node involvement (N0), peripancreatic nodal involvement (N +), and metastatic disease found during exploration (M1). The median overall patient survival for N0 status was 65 months (95% CI 37.8–90.2), 18 months (95% CI 15.7–20.3) for N + status, 9 months (95% CI 6.9–11.2) for PALN involvement, and 3 months (95% CI 1.6–4.4) for M1 status (*p* < 0.001) (Fig. 3c).

For PDAC only, the median overall patient survival for N0 status was 35 months (95% CI 4.2–65.8), 17 months (95% CI 15.6–18.4) for N + status, 9 months (95% CI 6.8–11.2) for PALN involvement, and 3 months (95% CI 1.6–4.4) for M1 status (*p* < 0.001) (Fig. 3d). A direct comparison of median overall survival between patients with PALN involvement and M1 disease demonstrated significantly decreased survival in patients with M1 disease (*p* < 0.001).

### Univariable and Multivariable Cox Regression Analysis in Patients with PALN Involvement

After univariable analysis, severe comorbidity (ASA grade 2 or higher) was a significant predictor for survival in patients with PALN involvement, with an HR of 3.139 (1.506–6.541; *p* = 0.001). Performing a pancreatoduodenectomy instead of a double bypass procedure in patients with PALN involvement yielded an HR of 1.660 (0.983–2.804; *p* = 0.058), whereas patients who did not undergo adjuvant chemotherapy had an HR of 1.565 (0.895–2.737; *p* = 0.116). Severe complications, defined as a Clavien–Dindo score of 3 or higher, yielded an HR of 1.273 (0.716–2.265; *p* = 0.412). After multivariate analysis, only severe comorbidity (ASA grade 2 or higher) was associated with shorter survival in patients with PALN involvement, with an HR of 3.121 (1.497–6.506; *p* = 0.002) (electronic supplementary Table 2).

## Discussion

In the present study, we have demonstrated that patients with PALN involvement who underwent a pancreatoduodenectomy had a median survival of 11 months, compared with 7 months in patients with PALN involvement who underwent a palliative double bypass procedure. Among a subset of PDAC patients with PALN involvement who received adjuvant chemotherapy, pancreatoduodenectomy was associated with a median survival of 13 months, compared with a median survival of 11 months for patients who underwent a double bypass procedure. Although a significant survival benefit was observed in patients who underwent pancreatoduodenectomy, this came at the cost of significantly increased morbidity, as illustrated by increased CCI scores, more severe complications, and a longer hospital stay, compared with patients who underwent a double bypass procedure.

The clinical value of routine PALN sampling has been investigated previously; 7^–^12 however, in these studies, all patients underwent pancreatoduodenectomy without routinely sampling of the PALN. Because of conflicting results and methodological imperfections, strong data to support routine PALN sampling and immediate frozen section analysis are lacking. However, the present study is the first to demonstrate the overall survival results and complication data of patients who underwent resection, as well as from a distinct group of patients in which resection was avoided, followed by palliative surgery.

The literature was recently reviewed in depth by van Rijssen et al. 18 Based on this review, we considered two recent publications of prospectively collected series of routine sampling of PALN, with conflicting results, to shape the current discussion. The first study by Schwarz et al. showed the results of a prospectively collected series with routine sampling of PALNs.^10^ In a 10-year period, 111 consecutive patients were included. Schwarz et al. found 12 patients to have involved PALNs after frozen section analysis, and another 5 patients after hematoxylin–eosin staining (11% and 15%, respectively). Median survival was 9.7 versus 28.5 months for positive and negative PALNs (*p* = 0.012). The second study, published by Nappo et al., included 135 consecutive patients in an 8-year period.^19^ Nappo et al. found involved PALNs in 15 (11%) patients. Median survival for PALN-positive patients was inferior to N0 but similar to that of N1 patients (32 vs. 69 vs. 34 months, respectively). In both studies, there was no clinical consequence of positive PALN findings.^10^^,^^19^ Both studies, and for that matter all other previous studies on this subject, have compared survival of resected PALN-positive patients with resected PALN-negative patients. We can therefore only conclude, on the basis of the previous studies, that positive PALNs are a marker of poor prognosis. However, the most important clinical question remains unanswered: should we avoid resection if positive PALNs are found, or not?

The present study may, at least in part, answer that question since this study shows both the results of resection and the results after an avoided resection followed by palliative bypass surgery. We compared both strategies where either strategy was ‘routine’. In cohort A, PALN involvement was considered a contraindication to resection and resection was avoided when positive PALNs were encountered by routine frozen section. Therefore, in this cohort, we demonstrated the outcomes after palliative treatment. On the other hand, in cohort B, PALNs were routinely sampled but not sent for immediate frozen section analysis, thereby showing the results after curative-intent resection.

Although the strategy regarding PALNs was different in both cohorts, the median overall survival of all patients who underwent pancreatoduodenectomy in cohort A was 21 months, compared with 18 months in cohort B (*p* = 0.238). After stratifying by PDAC only, median survival after pancreatoduodenectomy in cohort A was 17 months, versus 18 months in cohort B. Patients with PALN involvement generally did not undergo resection in cohort A, but did undergo a double bypass procedure. Therefore, a longer median survival was expected in cohort A because of a more favorable case selection of patients undergoing pancreatoduodenectomy. Several clinical and pathological characteristics between both cohorts were significantly different. After correcting for PDAC only, most characteristics were similar, with the exception of adjuvant chemotherapy. However, after a sensitivity analysis regarding patients with PDAC who received adjuvant chemotherapy, the results remained similar. The exact reason as to why a smaller proportion of patients in cohort A received adjuvant chemotherapy is unclear, however substantial variation of adjuvant chemotherapy rates for patients with pancreatic head carcinoma among different centers have been described.^20^^,^^21^

In the present study, patients with PALN involvement who underwent resection had a median overall survival of 11 months, compared with 7 months in patients where resection was avoided (*p* = 0.049). In a smaller subgroup of patients with PALN involvement who received adjuvant chemotherapy, the median overall survival was 13 months after pancreatoduodenectomy, compared with 11 months after a palliative bypass procedure (*p* = 0.033). Although the latter overall survival benefit may seem small (2 months) and was based on a small subset of patients, in the CONKO-001 trial the median overall survival benefit of adjuvant gemcitabine versus observation alone was also 2 months, but became the rationale for adjuvant chemotherapy worldwide.^22^

Patients with liver or peritoneal cavity metastases who also had PALN involvement were excluded to prevent an overestimated difference between the groups. However, higher morbidity and a longer hospital stay were seen in patients who underwent resection compared with a double bypass procedure. Furthermore, after univariable and multivariable Cox regression analysis in patients with PALN involvement, only severe comorbidity (ASA grade 2 or higher) was significantly associated with shorter survival in patients with PALN involvement. Therefore, the decision to perform a pancreatoduodenectomy with PALN involvement should be taken carefully in selected, fit patients because of limited survival benefit and an increased risk of morbidity compared with a double bypass procedure.

The ISGPS did not reach consensus regarding routine resection of PALNs due to variation in the literature and different expert opinions.^13^ Some consider PALN involvement as metastatic disease, while others see it as ‘normal’ lymph node involvement and thus N1. According to the 8th TNM classification, PALNs are extraregional nodes for both pancreatic and periampullary cancer. Our data show that the overall median survival of PALN involvement is significantly longer compared with M1 disease, but significantly shorter when compared with peripancreatic nodal involvement (Fig. 3).

Systemic chemotherapy is the main treatment for patients with locally advanced or metastatic pancreatic cancer. In a recent meta-analysis, patients with locally advanced pancreatic cancer treated with FOLFIRINOX had a median overall survival of 24 months—longer than that reported for patients with resected pancreatic cancer (stage I or II) treated with adjuvant gemcitabine in the ESPAC-3 trial.^23^^,^^24^ Neoadjuvant FOLFIRINOX could also benefit patients with PALN involvement. A minimally invasive approach is increasingly applied for pancreatic head resection and could be useful to determine PALN involvement prior to surgical resection. If positive PALNs are found, neoadjuvant chemotherapy might be considered in an attempt to improve overall median survival after pancreatoduodenectomy. Future research should assess if these patients might benefit from resection after FOLFIRINOX. Unfortunately, adequate preoperative evaluation of lymph node involvement beyond the peripancreatic chain is difficult since diagnostic accuracy of CT for assessment of extraregional lymph node metastases is poor. Furthermore, prior studies have demonstrated that it is also not possible to reliably determine lymph node status with endoscopic ultrasound during preoperative workup.^6^ Therefore, it is currently only possible to reliably determine PALN status during exploration with frozen section analysis.

Some limitations of the current study may be identified. First, the sample size of the study is limited, the reasons for which are partially due to the specific population studied. Patients with PALN metastasis resembled only one-fifth of the population who were selected for resection, which in turn is only one-sixth of patients who present with PDAC.^1^ Second, the retrospective design of the study has its known limitations. Complications were only assessed based on the available medical records. Major complications were surely noted, but possibly some minor complications have been missed. It was impossible to assess quality of life retrospectively because the majority of patients were deceased; however, quality of life is an important outcome measure, especially in the current population studied with such poor prognosis, and should be the subject of future prospective research initiatives.

## Conclusion

In patients with PALN metastases, pancreatoduodenectomy was associated with significant survival benefit compared with a double bypass procedure, irrespective of adjuvant chemotherapy, but with increased risk of complications. It is important to weigh the advantages of resection versus bypass against patient factors such as comorbidities, age, and clinical performance when positive intraoperative PALNs are found.

## Electronic supplementary material

Below is the link to the electronic supplementary material.

Supplementary material 1 (DOCX 14 kb)
