# Supplementary material for: How to Approach Para-Aortic Lymph Node Metastases During Exploration for Suspected Periampullary Carcinoma: Resection or Bypass?
Source: Ann Surg Oncol. 2020 Mar 10;27(8):2949–58. doi: 10.1245/s10434-020-08304-0 (PMC7334266; doi:10.1245/s10434-020-08304-0)
Supplement: Supplementary file 1 — Supplementary material 1 (DOCX 14 kb) [file 10434_2020_8304_MOESM1_ESM.docx]

**Supplementary table 1. Characteristics and postoperative outcomes of patients with positive para-aortic lymph nodes who underwent pancreatoduodenectomy (PD) versus double bypass procedure (DBP). All patients received postoperative chemotherapy.**

|  | **Resection (PD)**  **(*n* = 13)** | **Bypass (DBP) (*n* = 5)** | ***P-value*** |
| --- | --- | --- | --- |
| ***Characteristic*** |  |  |  |
| Age at surgery (years), *median (IQR)* | 64 (55-72) | 57 (46-68) | 0.387 |
| Sex: males, *n (%)* | 7 (53.8) | 4 (80.0) | 0.596 |
| ASA fitness grade, *n (%)* - Class I/II - Class III | 11 (84.6) 2 (15.4) | 4 (80.0) 1 (20.0) | 1.000 |
| Clavien-Dindo score ≥ 3, *n (%)* | 7 (53.8) | 0 (0.0) | 0.101 |
| CCI-score, *median (IQR)* | 33.5 (13.6-37.6) | 0 (0-10.5) | **0.004** |
| Time until discharge (days), *median (IQR)* | 11 (10-15) | 8 (7-11) | 0.117 |
| 30-day mortality, *n (%)* | 0 (0.0) | 0 (0.0) | 1.000 |
| 90-day mortality, *n (%)* | 0 (0.0) | 0 (0.0) | 1.000 |

Data are given in numbers with percentages (%) or medians with interquartile ranges (IQR). For comparison between two groups Mann-Whitney U test were used for continuous variables and for binary variables Chi squared test or Fisher’s exact test were used. Abbreviations: ASA, American society of anesthesiologists; CCI, comprehensive complication index.

**Supplementary table 2. Univariate and multivariate cox regression analysis of overall survival in patients with positive para-aortic lymph nodes**

| **Variable** | **Univariate analysis HR (95% CI)** | ***P*-value** | **Multivariate analysis HR (95% CI)** | ***P*-value** |
| --- | --- | --- | --- | --- |
| **Sex**  Female  Male | Ref  0.941 (0.562-1.576) | 0.817 |  |  |
| **ASA Score**  ASA I-II  ASA III-IV | Ref  **3.139 (1.506-6.541)** | **0.001** | Ref  **3.121 (1.497-6.506)** | **0.002** |
| **Surgical procedure**  Pancreatoduodenectomy  Double bypass procedure | Ref  **1.660 (0.983-2.804)** | **0.058** | Ref  1.651 (0.977-2.789) | 0.061 |
| **Clavien-Dindo score**  < 3  ≥ 3 | Ref  1.273 (0.716-2.265) | 0.412 |  |  |
| **Postoperative chemotherapy**  Yes  No | Ref  1.565 (0.895-2.737) | 0.116 |  |  |
| **Era of surgery**  2013-2016  Before 2013 | Ref  1.320 (0.786-2.218) | 0.294 |  |  |

Bold in univariate analysis indicates variables (p < 0.10) that were entered in multivariate analysis. Bold in multivariate analysis indicates statistical significance (p < 0.05). Abbreviations: ASA, American society of anesthesiologists.
